# Knowledge and Attitude of Human Monkeypox Viral Infection Among Healthcare Practitioners and Students in Saudi Arabia: A Cross-Sectional Study

**DOI:** 10.7759/cureus.45092

**Published:** 2023-09-12

**Authors:** Seham H Aljahdali, Wed O Albeshri, Sadeem S Allqmani, Yosra Z Alhindi, Sahar Elashmoony

**Affiliations:** 1 College of Pharmacy, Umm Al-Qura University, Makkah, SAU; 2 Pharmacology and Toxicology, Faculty of Medicine, Umm Al-Qura University, Makkah, SAU; 3 Clinical Pharmacy, Umm Al-Qura University, Makkah, SAU

**Keywords:** saudi arabia, pandemic, infection, virus, monkey pox, attitude, knowledge

## Abstract

Background: Human monkeypox (Mpox) is a viral zoonotic infectious disease occurs mostly in central and western Africa that can be transmitted to humans and animal. On July 2022, the World Health Organization declared the global Mpox outbreak, which considered as a huge health issue. The prevalence of Mpox in Saudi Arabia has been very low until now.

Aim: This research aims to assess knowledge and attitudes of healthcare practitioners and students toward human Mpox in Saudi Arabia.

Methods: A cross-sectional study conducted among healthcare practitioners and students in Saudi Arabia assessing knowledge, practice and attitudes towards Mpox infection. We conducted descriptive statistics for all variables.

Results: A total of 212 participants were included in the study. The majority of participants provided correct responses about the type of microorganisms that cause human Mpox infection and were aware about the low prevalence of the Mpox in Saudi Arabia, but they were not able to identify the correct number of cases in Saudi Arabia. About the symptoms of Mpox, participants showed good knowledge about the common symptoms like skin rash but poor knowledge about less common symptoms like lymph-node swelling. About 70% of participants were aware of the effectiveness of antiviral drugs to treat Mpox.

Conclusion: Gaps in knowledge were detected among participants. Therefore, increasing knowledge of Mpox by providing educational courses for healthcare practitioners and healthcare students is crucial.

## Introduction

Human monkeypox (Mpox) is a zoonotic infectious disease caused by a DNA virus, called the Mpox virus, belonging to the Poxviridae family. The first case appeared in 1970 in a child with smallpox-like symptoms. The World Health Organization (WHO) proclaimed the worldwide Mpox outbreak to be a public health emergency of international concern on July 23, 2022 [1]. Mpox infection was given its name in 1985. The WHO published the best practices for the naming of human infectious diseases in 2015. The aim of naming a new disease is to minimize unnecessary negative impacts of names on trade, tourism, travel, and animal welfare. The WHO stated that disease names may not include a class of animal or species. So, in November 2022, the WHO adopted Mpox as a new name for Mpox infection [2]. The danger of Mpox is currently modest worldwide, with the exception of the region of the European Union, where the risk is still high [3]. In Saudi Arabia, there are only eight confirmed cases until now. The first case was reported on July 14, 2022 in the capital Riyadh. The prevalence is very low but there is a risk since people from all over the world come to Saudi Arabia for the Hajj and Umrah [4]. Mpox Infection is transmitted to humans mainly from wild animals like monkeys and rodents and from one person to another by direct contact with the blood of infected animals. General symptoms include fever, rash, headache, lymphadenopathy, and myopathy, and respiratory symptoms include: sore throat and cough [5]. Mpox infection lasts between two and four weeks and is typically self-limiting. However, sensitive individuals, such as children and those with compromised immune systems, might occasionally develop serious complications [6]. The generation time was 12.5 days, but the mean incubation duration was 9.1 days. Depending on the severity of the symptoms and patient general health, physicians decide how to monitor symptoms, the need of isolation to an individual, and the urgent need to perform a contact tracing [7].

Currently, no drug has been approved by the US Food and Drug Administration (FDA) for the treatment of Mpox. However, tecovirimat, an antiviral drug that has been FDA approved for the treatment of smallpox, was used for the treatment of Mpox in the US under the FDA-regulated Expanded Access Investigational New Drug (EA-IND) mechanism [8]. No data is available on the effectiveness of tecovirimat in treating people with Mpox. However, research on animals has demonstrated the efficacy of tecovirimat in treating illnesses brought on by orthopoxviruses [9].

One of the most challenges that faced healthcare practitioners to prevent the re-emergence of Mpox was the lack of knowledge about Mpox infection. Therefore, healthcare practitioners and students must be knowledgeable and ready for Mpox cases [10]. This research aims to evaluate the knowledge and attitude toward Mpox among healthcare practitioners and healthcare students.

## Materials and methods

Study design

This study was carried out as a cross-sectional study for healthcare practitioners and students to assess their knowledge and attitude toward Mpox infection by using an electronic questionnaire (google form).

Validity and reliability

To make sure that the survey questions are valid and reliable we distributed the same questions to 10 healthcare workers (HCWs) and 10 healthcare students outside the participant group. Data was then collected and analysed with good statistical power.

Study population

Inclusion Criteria

The study sample comprised first-line healthcare practitioners likely to be involved in the initial management of Mpox cases, such as public health medical professionals, general practitioners, occupational physicians, Pharmacists, Nurses, and healthcare students.

Exclusion Criteria

Exclusion criteria included any one aged younger than 18 years old, non-HCWs, and non-healthcare students.

Data-collection tools and process

Data was collected through an electronic questionnaire in English, and some questions adapted from published studies [11, 12]. Some questions were modified and added to meet the research objectives. The study was conducted using a Google form template and distributed to the target population by using social media (WhatsApp, Telegram, Twitter). The questionnaire contains four sections: consent form, sociodemographic data, assessment of the knowledge, and attitude towards Mpox. The first part the survey is the consent form, which enable participants to have the freedom either to participate or not in this study by answering the questions. The second section addressed the sociodemographic data such as age, gender, level of education, nationality, college of healthcare students, occupation of healthcare practitioners, years of work experience, and region of work or study. The third section contains a close-ended question to assess knowledge of human Mpox infection, consisting of 13 questions, a the last section contains a close-ended question to asses the attitude toward human Mpox infection consisting of 12 questions with the option for an answer either (agree, neutral, or disagree). All questions were mandatory to respond to prevent nonresponding bias.

Ethics and confidentiality

This study is ethically approved and reviewed by Umm Al-Qura University Institutional Research Board (IRB). The approval number: (HAPO-02-K-012-2022-11-1256). Survey responses were collected in an anonymous fashion, no private or identifying information from participants was collected, and all responses' confidentiality was maintained and used only for research purposes.

Statistical analysis

RStudio (R version 4.1.1) was used for data analysis. Categorical variables were expressed as frequencies and percentages, whereas numerical variables were presented as the median and interquartile range (IQR). A knowledge score was calculated by summing up 12 knowledge items. Each correct response was assigned 1; therefore, the knowledge score ranged between 0 and 12, where a higher score indicated better knowledge. Factors associated with knowledge were assessed by constructing a multivariate linear regression model. The knowledge score was used as a dependent variable, and the demographic variables were entered as independent variables (gender, nationality, age, level of education, and region of work). The independent variables were entered using a forward stepwise selection method. Results were expressed as beta coefficients and 95% confidence intervals (CIs). Statistical significance was indicated by p < 0.05.

## Results

Demographic and academic/occupational characteristics

In the current study, we initially received 219 responses on the online platform. However, one response was excluded for a respondent who declined to participate, and six responses were from participants who were not working in the healthcare niche. Therefore, a total of 212 responses were ultimately analyzed.

The majority of respondents were female (82.1%) and Saudi (97.2%), and 96.7% of them were aged 18 to 29 years. A great proportion of respondents were working or studying in the western region (80.2%) (Table 1). Students represented 80.1% of the sample, while interns and HCWs constituted 10.0% and 9.9% of the participants, respectively. Focusing on students (169), respondents were primarily belonging to the College of Pharmacy (55.6%) and College of Medicine (16.0%). Six students belonged to other colleges, and these included three students from Health Information and Management Technology (3.3%), two students from Public Health (1.1%), and one student from Clinical Nutrition (0.5%). As from the regression analysis results, there was no significant differences in knowledge between pharmacy and medicine students. Concerning HCWs (n=21), pharmacists represented almost one-third of the participating HCWs (38.1%), followed by physicians (19.0%). Of note, 38.1% of HCWs had ≤ 1 year and 2-5 years of experience (for each category), 9.5% had 6-10 years, and 14.3% had higher levels of experience (Table 1).

**Table 1:** Demographic and academic/occupational characteristics ^*^ The variable has one missing value. ¥ Descriptive statistics are based on 21 HCWs. HCW: Healthcare worker

Parameter	Category	N (%)
Gender	Male	38 (17.9%)
	Female	174 (82.1%)
Nationality	Saudi	206 (97.2%)
	Non-Saudi	6 (2.8%)
Age	18 to 29	205 (96.7%)
	30 to 49	6 (2.8%)
	50 to 69	1 (0.5%)
	≥70	0 (0.0%)
Level of education^*^	Student	169 (80.1%)
	Intern	21 (10.0%)
	Specialist	10 (4.7%)
	Resident	5 (2.4%)
	Consultant	3 (1.4%)
	Other	3 (1.4%)
Region of work/study	Western	170 (80.2%)
	Eastern	20 (9.4%)
	Central	10 (4.7%)
	Northern	9 (4.2%)
	Southern	3 (1.4%)
Years of work experience of HCWs^¥^	≤1 year	8 (38.1%)
	2 to 5 years	8 (38.1%)
	6 to 10 years	2 (9.5%)
	>10 years	3 (14.3%)

Awareness and knowledge about human Mpox virus:

Generally, 175 participants were aware of human Mpox virus, representing 82.5% of the sample (95% CI, 76.7-87.3). The majority of participants provided correct responses to the fact that Mpox is a virus (94.3%) and that a skin rash is a symptom of the infection (74.5%). More than half of the participants indicated a correct response for the low prevalence of the human Mpox infection in Saudi Arabia (53.3%) and that the virus is not exclusively transmitted from animals to the human (59.4%). However, less than half of the respondents correctly identified the number of confirmed cases of the infection in Saudi Arabia (33.0%) and lymph node swelling as a symptom of the infection (43.4%). From Table 2, it is apparent that around 40.6% of the respondents had good knowledge about the neurological symptoms that were caused by Mpox. More details about the correct responses to knowledge items are demonstrated in Table 2.

**Table 2:** Participants' response to knowledge items *An asterisk indicates a correct response Mpox: Monkeypox

Parameter	Category	N (%)
Mpox infection is caused by:	Virus*	200 (94.3%)
	Bacteria	3 (1.4%)
	Fungi	6 (2.8%)
	None of the above	3 (1.4%)
The prevalence of human Mpox is high in Saudi Arabia.	True	12 (5.7%)
	False*	113 (53.3%)
	Do not know	87 (41.0%)
Number of confirmed cases of human Mpox in Saudi Arabia	Less than 5*	70 (33.0%)
	Less than 10	78 (36.8%)
	Less than 50	42 (19.8%)
	Less than 100	22 (10.4%)
Mppox is transmitted from animal to person ONLY.	True	20 (9.4%)
	False*	126 (59.4%)
	Do not know	66 (31.1%)
Mpox and smallpox have similar signs and symptoms.	True*	61 (28.8%)
	False	32 (15.1%)
	Do not know	119 (56.1%)
Mpox and chickenpox have similar signs and symptoms.	True	61 (28.8%)
	False*	43 (20.3%)
	Do not know	108 (50.9%)
Symptoms of human Mpox infection include swelling of lymph node.	True*	92 (43.4%)
	False	12 (5.7%)
	Do not know	108 (50.9%)
Symptoms of human Mpox infection include skin rashes.	True*	158 (74.5%)
	False	4 (1.9%)
	Do not know	50 (23.6%)
Mpox leads to neurological and psychiatric problems in some patients.	True*	86 (40.6%)
	False	14 (6.6%)
	Do not know	112 (52.8%)
Both Covid-19 and human Mpox infection mainly can affect:	Gastrointestinal system	10 (4.7%)
	Respiratory system*	152 (71.7%)
	Cardiovascular system	50 (23.6%)
	All of the above	0 (0.0%)
To treat human Mpox, which of the following can be used:	Antiviral drugs*	150 (70.8%)
	Antibiotic drugs	62 (29.2%)
	Antifungal drugs	0 (0.0%)
	Drugs not effective in treating Human monkeypox infection	0 (0.0%)
The vaccine used for smallpox is effective in preventing Mpox infection.	True*	60 (28.3%)
	False	39 (18.4%)
	Do not know	113 (53.3%)

Knowledge score and the associated factors

Based on the 12 items of knowledge, the median (IQR) knowledge score was 5.0 (3.0 to 6.0). The score ranged between 1 and 9. Based on the regression analysis, participants’ knowledge was independently associated with age, where participants aged 30 to 49 (beta = 2.09, 95% CI 0.12-4.05, p = 0.038) and 50 to 69 (beta = 4.69, 95% CI 0.82-8.55, p = 0.018) had higher scores of knowledge (Table 3).

**Table 3:** Results of the regression analysis for the predictors of participants' knowledge about human Mpox virus HCW: Healthcare worker

Parameter	Category	Beta	95% CI	p-value
Gender	Male	0.1	0.05	
	Female	-0.13	-0.88, 0.62	0.735
Nationality	Saudi	0.02	0.022	
	Non-Saudi	-0.64	-2.33, 1.06	0.460
Age	18 to 29	0.011	0.1	
	30 to 49	2.09	0.12, 4.00	0.038
	50 to 69	4.69	0.82, 8.55	0.018
Level of education	Student	0.11	0.03	
	Intern	0.03	-0.85, 0.90	0.954
	HCW	0.02	-0.86, 0.89	0.972
Region of work/study	Western	0.02	0.4	
	Eastern	0.71	-0.19, 1.60	0.121
	Central	-0.62	-1.93, 0.70	0.358
	Northern	0.15	-1.26, 1.56	0.832
	Southern	0.11	-2.08, 2.30	0.920

Participants’ attitudes towards Human Mpox virus

The majority of participants agreed that they were confident about the ability of the Saudi Ministry of Health (MOH) and the local population to control the local spread of Mpox (87.3%). Additionally, 82.1% of them agreed that it is important for students to learn about the infection, and 81.6% of them confirmed the necessity of providing courses about the infection to healthcare practitioners. On the other hand, around less than half of the participants did not have any concerns about the possibility of Mpox to spread and become a global pandemic (42.0%). Moreover, they denied any suspicions regarding the possibility of new prevention and control measures of Mpox occuring in Saudi Arabia (41.5%). They also denied any possibility of increase in the number of Mpox cases in Saudi Arabia despite the seasons of crowdedness like the Hajj and Umrah (41.5%) (Table 4).

**Table 4:** Percentages of participants' responses to the attitude's items

Percentage	Attitude
84.00%	Confident about Saudi control
82.10%	Important to educate about the virus
81.60%	Courses
42%	Spreading
41.50%	Suspicions
41.50%	Cases during the Hajj

## Discussion

Mpox is threatening public health in several countries. Globally, the outbreak involved 125 countries, but only 29 of them have reported Mpox historically [12]. The prevalence of Mpox in Saudi Arabia is very low until now, but a higher risk is suspected because Saudi Arabia is a religious country where people from all over the world come for the Hajj and Umrah. One of the challenges that faced healthcare practitioners in prevention of the re-emergence of Mpox globally was the lack of knowledge about Mpox infection [13]. Therefore, in order to respond to the requirement of Mpox outbreak, healthcare practitioners and students must be knowledgeable about the sign and symptoms, prevention measures, available treatment options, and vaccinations for Mpox.

In the current study, a total of 212 participants were included out of 219, and the majority of participants provided correct responses about the type of microorganisms that cause human Mpox infection, which is a virus, these results were comparable to a previous study that was conducted in Indonesia. This study aimed to determine general practitioners' knowledge regarding Mpox, and majority of participants (96.6%) knew Mpox is a virus [14]. In the current study, majority of participants were aware of the low prevalence of Mpox in Saudi Arabia, but only small proportion identified the correct number of cases in Saudi Arabia. In November 2022, when the survey was published and the data collected, the number of confirmed cases of Mpox in Saudi Arabia was less than five. Recently, in April 2023, according to the Centers for Disease Control and Prevention (CDC), there were eight confirmed cases of Mpox in Saudi Arabia [15].

Both Mpox and smallpox are poxviruses. They are similar to each other in many aspects, but Mpox symptoms are milder and the mortality rate is lower compared with smallpox. Mpox is not related to chickenpox, which is caused by herpesvirus varicella zoster [16]. The data of the present study showed poor knowledge about the similarity of the signs and symptoms of Mpox and smallpox, as only 28.2% of participants answered correctly. These findings correspond with a study of Saudi physicians where more than 57.9% of physicians said that signs and symptoms of smallpox and Mpox are not like each other [17]. The current results showed poor knowledge about the differences between Mpox and chickenpox, as only 20.3% answered correctly.

Participants showed good knowledge about the common symptoms of Mpox, like skin rash, but poor knowledge about less common symptoms like lymph node swelling. Mpox causes neurological and psychiatric complications including headache, confusion, myalgia, depression, anxiety, and neuropathic pain [18]. Around 40.6% of the respondents had good knowledge about the neurological and psychiatric symptoms that were caused by Mpox.

Although all antivirals used for Mpox still have not been approved by FDA for this indication, these antivirals include tecovirimat, cidofovir, and brincidofovir. However, not all patients with Mpox infection are treated with these antiviral drugs [19]. According to the CDC, most of Mpox cases are mild and self-limited. These cases are mainly controlled by supportive care and symptomatic treatment only. Different drugs can be used to treat Mpox symptoms including paracetamol, non-steroidal anti-inflammatory drugs (NSAIDs), steroids, and local anesthetics to relieve pain. About 70% of participants in our research were aware of the effectiveness of antiviral drugs to treat Mpox. In a similar study conducted among clinicians in Ohio, USA, almost half of the participants (52%) were aware of the availability of effective drugs against Mpox [20]. Also, a recent study was conducted among preclinical dental students in Malaysia. They showed poor knowledge of treatment options only 22.9% of them knew that paracetamol can be used for treating symptoms of Mpox [21]. 

The vaccine used for smallpox eradication is now used for Mpox prevention (JYNNEOS vaccine), previous meta-analysis results demonstrated that patients vaccinated against smallpox had fewer adverse effects and complications relating to Mpox compared with vaccine-naïve patients [22]. The current results showed poor knowledge about the Mpox vaccine. Only 28.3% of participants answered correctly. Compared to Alshahrani and colleagues’ results, there were approximately 70% physicians who had a good knowledge of the Mpox vaccine [1]. This mass difference in knowledge may be due to the difference in the targeted population. In the current study, most participants (80%) are students. Alshahrani et al. targeted physicians only. A possible explanation could be that physicians had better scientific backgrounds and more medical training than students. 

In the current study, less than half of the participants (41.5%) disagreed regarding adopting adequate prevention and control measures. Previous research found unfavorable attitudes toward Mpox immunization. For instance, a survey study conducted among HCWs in Czech Republic revealed that only 9% of the study participants had consented to receive the Mpox immunization [23]. On the contrary, a survey study among medical workers in China showed a positive attitude toward Mpox vaccination [24]. Previous studies revealed that different nations throughout the world have varying levels of knowledge and attitudes toward Mpox immunizationn [25-28].

A previous study conducted in Saudi Arabia stated the importance of the guidelines and protocols implementation to manage Mpox [24]. In June 2022, Saudi MOH published a protocol for Mpox infection to facilitate the selection of the best therapeutic option based on the best available evidence with respect to clinical judgment and patient preferences. In the current results, the majority of participants (87.3%) agreed that they were confident in the ability of MOH and the local population to control the local spread of Mpox. 

Religious festivals attract millions of pilgrims from worldwide which consider a potential risk for the transmission of infectious diseases. Mass gatherings like the Hajj and Umrah could compromise the overall health system in Saudi Arabia [23]. The current results showed that less than half of the participants disagreed about the possibility of an increase in Mpox cases in Saudi Arabia with the Hajj and Umrah.

Regarding demographic and academic/occupational characteristics of participants association with knowledge, the highest percentage of participants were in the 18-29 age group and were students, which may partly explain the statistical results. Results showed no significant association between knowledge and level of education, gender, nationality, or region of participants. Significant difference was clearly shown in ages as participants older than 30 years old had better knowledge than participants younger than 30 years old [24]. On the other hand, a similar study among university students in Pakistan showed that the type of academic degree, discipline, and region of respondents was significantly associated with the overall knowledge of Mpox disease [15].

Gaps in knowledge in some disease aspects were detected in the current study and other similar recent studies, and observable knowledge gaps in some disease aspects including treatment and vaccination were reported among Saudi physicians [16]. In another study among HCWs in Lebanon, physicians have shown a better level of knowledge, but knowledge gaps were still noticeable [23].

One of the limitations of this current study that it is a cross-sectional type with limited data. Another is the short period of time taken to collect the data. Finally, there are zero cases of monkeypox in Saudi Arabia. Thus, keeping in mind that comparing our survey results from participant from Saudi Arabia to other countries that had been contacted with this virus will be of great value to the literature.

## Conclusions

In conclusion, gaps in knowledge for some disease aspects among participants were observed, particularly with regard to the number of Mpox cases, vaccinations, similarities, and differences between Mpox and other infectious diseases like smallpox and chickenpox. Therefore, increasing knowledge of Mpox by providing courses for HCW and academic courses for healthcare students is crucial, which is a key factor to enhance their ability to reduce illness burden and to respond well to any future Mpox cases.

Future research should explore the effectiveness of targeted educational interventions in improving the knowledge and attitudes related to Mpox, as well as the potential impact of these interventions on the occurrence and management of Mpox outbreaks. Furthermore, qualitative studies may provide additional insights into the barriers and facilitators of Mpox prevention and management.
